# Wrist‐worn accelerometers: Influence of decisions during data collection and processing: A cross‐sectional study

**DOI:** 10.1002/hsr2.1810

**Published:** 2024-01-10

**Authors:** Helén Eke, Stephanie E. Bonn, Ylva Trolle Lagerros

**Affiliations:** ^1^ Department of Medicine (Solna) Clinical Epidemiology Division, Karolinska Institutet Stockholm Sweden; ^2^ Center for Obesity, Academic Specialist Center, Stockholm Health Services Stockholm Sweden

**Keywords:** accelerometry, actiGraph, GGIR, methods, physical activity, sedentary behavior

## Abstract

**Background and Aims:**

Accelerometers collect data in an objective way, however, a number of decisions must be done during data collection, processing and output‐interpretation. The influence of those decisions is seldom investigated, reported, or discussed. Herein, we examined the influence of different decisions on the outcomes: daily minutes of moderate‐to‐vigorous physical activity (MVPA), inactivity and light physical activity (LPA).

**Methods:**

In total, 156 participants wore an accelerometer (ActiGraph wGT3X‐BT) on their nondominant wrist for 7 days. Data collection was conducted from February 2017 to June 2018. Data was processed using the R‐package GGIR and default settings were compared to by‐the‐literature‐suggested options. The output was examined using paired *t*‐tests.

**Results:**

When comparing two commonly used MVPA‐cut‐points, default and Hildebrand et al. we found a marginal difference (0.4 min, 1.0%, *p* < 0.001) in MVPA/day. When no bout criteria for MVPA/day was applied, MVPA/day was twice as high as bouted MVPA/day. Further, when we changed the epoch‐length from 5 to 1 s, statistically significant changes were seen for MVPA/day (−6.6 min, 19%, *p* < 0.001), inactivity/day (−22 min, 3.0%, *p* < 0.001) and LPA/day (28 min, 81%, *p* < 0.001).

**Conclusion:**

Decisions made during data processing of wrist‐worn accelerometers has an influence on the output and thus, may influence the conclusions drawn. However, there may be situations when these settings are changed. If so, we recommend examining if the variables of interest are affected. We encourage researchers to report decisions made during data collection, processing and output‐interpretation, to facilitate comparisons between different studies.

## 1 INTRODUCTION

Technical innovations have made objective assessments of physical activity possible and advanced research within the field of public health and epidemiology. Wearables, such as accelerometers, are available on a larger scale than before and feasible to use in both large‐scale epidemiological studies as well as in standard health care.

The early versions of accelerometers were hip‐worn and uniaxial.^1^ Data was processed simultaneously as it was collected, using brand‐specific methods,^2^ which resulted in a lack of awareness about the actual processing algorithms utilized.^3^ Today's accelerometers are triaxial.^1^ Raw data is stored in the device, and data processing is done afterwards.^1^ Further, wrist‐worn, instead of hip‐worn, accelerometers are becoming more common, since this increases compliance and enables sleep‐tracking.^1^, ^2^ A wrist‐worn accelerometer collects more acceleration‐data compared to a hip‐worn, though some of the additional data collected is only related to random wrist movements.^1^ Therefore, data collected using wrist‐worn accelerometers must be processed differently from hip‐worn accelerometers.

Even though accelerometers collect data in an objective way, a number of decisions must be made by the researcher, both during data collection, processing and output‐interpretation.^3^ This includes decisions regarding: sampling frequency, epoch‐length, definition of and how to handle nonwear time, cut‐points for the physical activity levels of interest, and which of the possibly hundreds of output‐variables to use.^1^, ^4^ Since these decisions can have a major influence on the final output, it is important that researchers apply them in a well‐thought‐out way. However, the field of accelerometry is not yet standardized and many do not report or justify the reasons for the key methodological choices made during data collection and processing when presenting their final results.^5^

We collected accelerometer‐data in a clinical study using a wrist‐worn accelerometer, but found no protocol or exhaustive guide for processing and analyzing this type of accelerometer‐data.

Therefore, the aim of this paper is to evaluate the influence of different decisions during data collection, processing and output‐interpretation, using wrist‐worn accelerometers (ActiGraph wGT3X‐BT). Also, by illustrating the influence that these decisions can have on the output, we want to highlight the importance of reporting these. Here we present results from analyses of data from accelerometer measurements performed within the trial, where we used default settings and compared them to commonly used options from the literature.

## 2 MATERIAL AND METHODS

### 2.1 Study design

This study is based on baseline data from a randomized controlled trial comprising 181 participants with diabetes type 2, recruited from February 2017 to June 2018 at primary care centers and one specialist center in Stockholm, Sweden. Inclusion criteria were: having a diagnosis of diabetes type 2, at least 18 years old, ability to communicate in Swedish, and having and being able to use a smartphone. Exclusion criteria were: not being able to walk. Details on the design can be found elsewhere.^6^ Herein, we used accelerometer‐data from baseline measurements, that is, before the intervention took place. The study was approved by the Regional ethical review board, Stockholm, Sweden (2016/2041–31/2; 2016/99–32; 2017/1406–32), and performed in accordance with the declaration of Helsinki. All participants provided written informed consent before enrollment.

### 2.2 Measurements

Participants were asked to wear the ActiGraph wGT3X‐BT accelerometer for all hours on their nondominant wrist, starting at 4 p.m. the same day, and removing it at 8 a.m. after seven full days. Data was collected at a sampling frequency of 80 Hertz (Hz), that is, 80 measurements per s.^7^

### 2.3 Accelerometer‐data processing using GGIR

Data processing to convert raw accelerometer‐data into output on physical activity was performed using the open‐source R‐package GGIR^4^ version 2.0‐0 (released 2020), R version 3.6.1 and RStudio version 1.2.5019. Raw data from each accelerometer was downloaded to a GT3X‐file by using the ActiGraph's brand‐specific program ActiLife version 6.13, and loaded into R/RStudio.

GGIR consists of five parts (part1−part5). The first part includes averaging raw acceleration‐data over epochs, and aggregation through application of Euclidean norm minus one (ENMO). Negative values are rounded up to zero.^8^ Next, an autocalibration function adjusts for calibration errors and replaces unreliable signals.^9^, ^10^ This is done using local gravity retrieved by the defined time zone of the measurement. Inaccurate data is replaced with averaged data from the same time the other measured days. Further, data collected before the first and after the last midnight is excluded to obtain seven complete days, using the setting *strategy*.^10^

Nonwear time was defined using default settings, that is, in bouts of at least 1 h (four consecutive 15‐min blocks).^8^ The nonwear time was replaced with the averaged activity the same time‐period the other measured days, similar to the autocalibration function.^8^ With GGIR you can chose to either derive data from wake‐up to wake‐up, or midnight to midnight.^10^ We defined the time‐window as midnight to midnight, to retrieve data summarized over 24 h.

Output on physical activity was produced in two steps, that is, GGIR part2 and part5.^8^ The output was summarized in absolute numbers as milli *gravity* (m*g*, 1 mg = 0.00981 m·s^−2^), and as time spent performing activities of different intensities, by using the average acceleration per epoch.^8^ The output on moderate‐to‐vigorous physical activity (MVPA)/day was produced in both part2 and part5, while output for inactivity/day and light physical activity (LPA)/day was only produced in part5.

To capture not only sporadic movements but also sustained activity, we obtained data on bouted variables for MVPA, LPA, and periods of inactivity. For MVPA, bouts for sustained activity was defined as at least 1 min with an 80% criterion,^11^ that is, 80% of the included epochs had to be equal to or above the MVPA‐cut‐point.^8^ Further, inactivity was defined as bouts of at least 10 min with 90% criterion and LPA as bouts of at least 1 min with 80% criterion.^8^ Default GGIR cut‐points was applied for inactivity (<40 m*g*) and LPA (40−100 m*g*).^8^ Due to bout criteria and weighting of variables, the output did not add up to 24 h.

### 2.4 Testing of settings

Default settings were identified and compared to one or two commonly used options from the literature. These settings were chosen after the data collection, but before the data processing and analysis. Settings were cut‐points for MVPA: 100,^8^ 100.6,^12^ or 110 m*g*,^13^ epoch‐length: 1,^12^ 5,^8^ or 60 s,^1^, ^14^ the definition of a valid day: 12, 14, or 16 h,^1^, ^15^ and valid week: 3 or 4 days.^1^, ^15^ Further, we used data extracted from GGIR part2 or part5, variables with a bout criterion or not, and weighted or plain variables. Default settings were applied to all parameters except the one that was tested. Our main outcome was minutes of MVPA/day, but for further comparisons, minutes of inactivity/day and LPA/day were also extracted.

### 2.5 Statistical analysis

Descriptive statistics are presented as mean values with standard deviations (SD). Two‐tailed paired *t*‐tests were used to examine any difference between output of the default setting and each alternative setting. Results are presented with mean values and *p* values. Spearman rank's test for correlation examined associations between number of valid days and minutes of MVPA/day, inactivity/day and LPA/day, respectively. These results are presented with the correlation coefficient (*r*) and corresponding *p* value. Participants that fulfilled the default inclusion criteria were included in the analysis, that is, had used the accelerometer for at least 16 h for 4 days (including at least 1 weekend day). All reported *p* values were two‐sided and *p* < 0.05 were considered statistically significant. Statistical analyses were performed using STATA 14.2.

## 3 RESULTS

**Figure 1:**
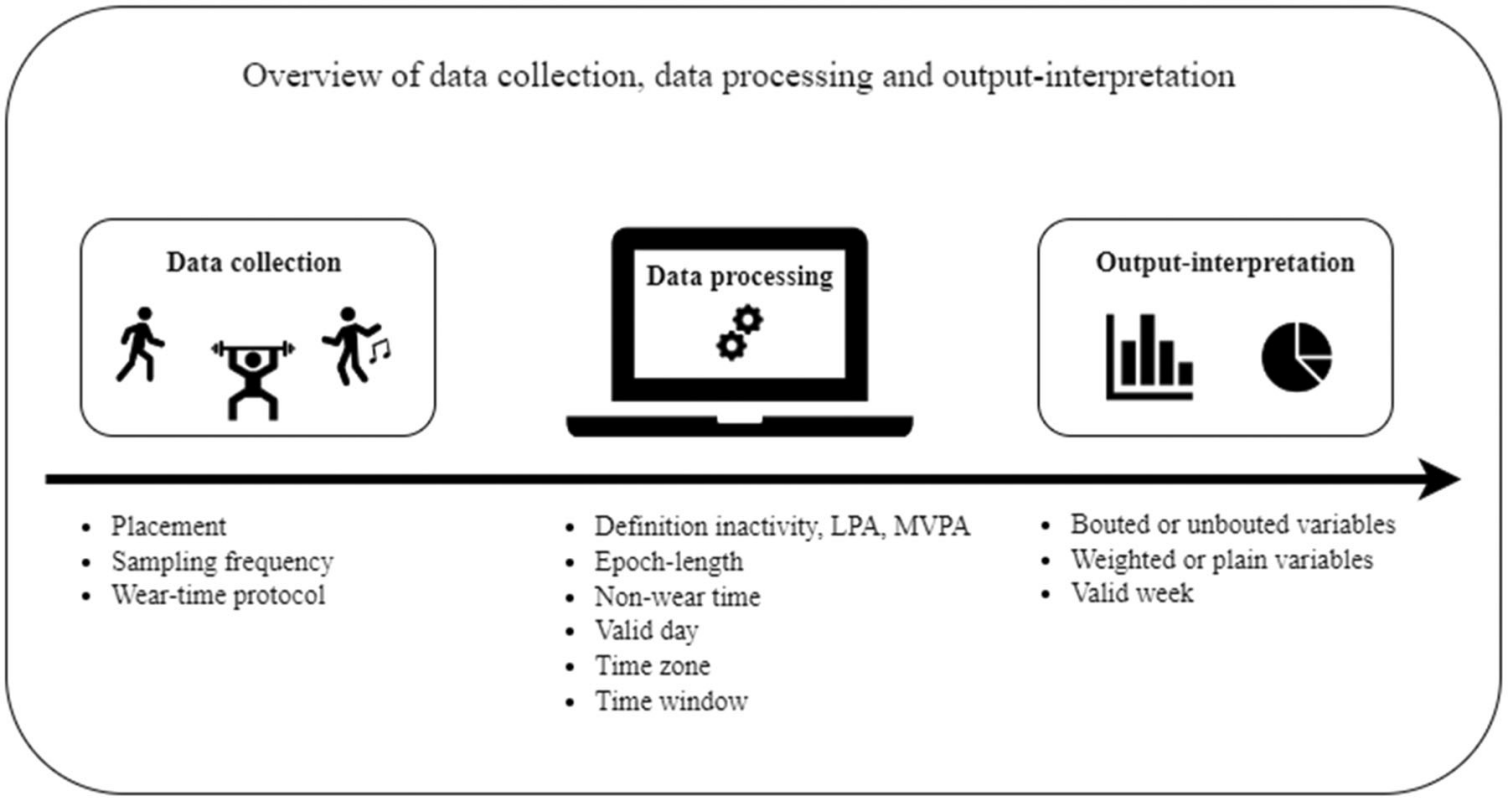
An overview of the process of collecting, processing and interpreting accelerometer‐data collected using a wrist‐worn accelerometer. LPA, light physical activity; MVPA, moderate‐to‐vigorous physical activity.

In total, 156 participants provided accelerometer‐data that fulfilled the inclusion criteria for accelerometer‐data processing. Participants were 66% male and had a mean age of 60 (SD 11) years. When using default settings for accelerometer‐data processing, an average of 33.9 (SD 26.5) min were spent on MVPA/day, while 698 (SD 179) min were spent inactive/day and 34.7 (SD 25.9) min were spent on LPA/day. Our decisions based on our results are also summarized in Table 1. Table 2 comprises practical tips gathered when accelerometer‐data was collected and processed within this trial. Figure 1 shows an overview of the process of collecting, processing and interpreting accelerometer‐data.

**Table 1:** Summary of decisions during data collection, processing and output‐interpretation.

Decision		Reason
*Data collection*		
1.	Placement: Nondominant wrist	Most common
2.	Wear‐time protocol: All hours, 7 days	Most common
3.	Sampling frequency: 100 Hz	Highest available
*Data processing*		
4.	Time zone: for example, Europe/Stockholm	Place of data collection
5.	MVPA‐cut‐point: 100 m*g*	Default
6.	Epoch‐length: 5 s	Default
7.	Valid day: 16 h	Default
8.	Nonwear definition: 4 × 15 min	Default
9.	Remove extra time: Use the function *strategy*	Remove time before/after the measurement
10.	Time‐window: Midnight to midnight and/or waking time to waking time	Use the first for a 24‐h window and latter to study sleep
11.	Examine certain time points: Use the function *qwindow*	Use if certain time points are of interest
*Output‐interpretation*		
12.	Data from part2 or part5: Part5	Most common
13.	Valid week: 4 days including ≥ 1 weekend day	Most common
14.	Bout criterion for MVPA‐output: ≥1 min with 80% of epochs equal to/above MVPA‐cut‐point	Remove activity due to random wrist movements
15.	Weighted or plain variables: Weighted	Eliminate effects due to different wear‐time

Abbreviations: Hz, Hertz; MVPA, moderate‐to‐vigorous activity; m*g*, milli *gravity*.

**Table 2:** Practical tips.

1.	To facilitate good compliance—equip the accelerometers with adjustable, elastic wrist‐bands, since the wrist circumferences may vary.
2.	When extracting the raw data from ActiGraph‐accelerometers, keep all GT3X‐files in the same folder. This enables ActiLife to extract all files simultaneously.
3.	Do not keep dates and timestamps when the files are extracted from ActiLife as GGIR cannot handle these. (The start date and start time is already automatically kept elsewhere in the file header, i.e., no information is lost.)
4.	If you are not familiar with GGIR or R/RStudio—see the tutorials below.
	*GGIR tutorial*—*installation and getting started* (https://youtu.be/S8YPTrYNWdU)
	*Analyze accelerometer data using GGIR* (https://youtu.be/RuFBCAqFJ2M)
5.	If you encounter problems or have questions regarding your data processing—join the google group “R‐package GGIR” where developers and users can connect.
6.	If you are using ActiGraph‐accelerometers and are unsure whether there is wear‐time on a certain file, and you are not that familiar with GGIR, it can be checked using ActiLife. Use the tabs “wear time validation” and “scoring.”

### 3.1 MVPA‐cut‐point

We found that MVPA/day was statistically significantly lower by on average 0.4 min (1.0%, *p* < 0.001) when the cut‐point was changed from default 100 m*g* ^8^ to 100.6 m*g* Hildebrand et al.^12^ When the suggested cut‐point for the dominant wrist 110 m*g* by Migueles et al.^13^ was applied, minutes of MVPA/day was statistically significantly lower by on average 6.2 min (18%, *p* < 0.001) in our data.

### 3.2 Epoch‐length

When the default epoch‐length of 5 s^8^ was changed to 1 s, average MVPA/day was statistically significantly lower by 6.6 min (19%, *p* < 0.001). Further, less time was classified as inactivity/day (−22 min, 3.0%, *p* < 0.001) and more time was classified as LPA/day (28 min, 81%, *p* < 0.001), compared to when the default 5 s epoch was applied. Instead, with a 60 s epoch, MVPA/day was statistically significantly higher by on average 19 min (57%, *p* < 0.001) compared to when the default epoch‐length was applied. Also, less time was defined as inactivity/day (−21 min, 3.0%, *p* = 0.004) as well as LPA/day (−157 min, 450%, *p* < 0.001).

### 3.3 Valid day

A valid day is commonly defined as 16 h, that is, two thirds of the day. In our data, the sample size increased by 3.0% (*n* = 5) and 5.0% (*n* = 8) when we changed the definition of a valid day from 16 h to 14 h and 12 h, respectively. Furthermore, MVPA/day became 0.08 min (0.28%) respectively 0.16 min (0.48%) higher, although these changes were not statistically significant (*p* = 0.35 and *p* = 0.15). LPA/day did not change statistically significantly when the criteria changed from 16 h to neither 14 h (−0.21 min, 0.61%, *p* = 0.27) nor 12 h (−0.47 min, 1.3%, *p* = 0.08). For inactivity/day, small, but statistically significant, changes were seen when the definition of a valid day changed from 16 h to 14 h (−4.0 min, 0.57%, *p* = 0.009), and to 12 h (−4.8 min, 0.68%, *p* = 0.005).

### 3.4 Valid week

A valid week is often defined as 4 days, including at least 1 weekend day.^1^, ^15^ When we changed the definition of a valid week to 3 days (including at least 1 weekend day), our sample size increased by 1.3% (*n* = 2). Those who did not fulfill the default criterion for a valid week, compared to those who did not, had on average 8.4 [SD 6.6] min lower MVPA/day, but this was not statistically significant (*p* = 0.21). Further, we found no correlation between number of valid days and minutes of MVPA/day (*r* = 0.10, *p* = 0.20), inactivity/day (*r* = −0.14, *p* = 0.06) or LPA/day (*r* = −0.007, *p* = 0.93), respectively.

### 3.5 GGIR part2 and part5

MVPA/day was on average 33.9 (SD 26.5) min when retrieved from GGIR part5, which is developed to improve accuracy between versions of the package. In contrast, we found that MVPA/day obtained from GGIR part2, which is developed to be consistent between different versions of the package, was on average 29 min higher (85%, *p* < 0.001).

### 3.6 Bouted MVPA‐variables

By default, a bout for consistent MVPA is defined as at least 1 min, where at least 80% of the included epochs must be equal to or above the MVPA‐cut‐point.^8^, ^11^ We found that MVPA/day doubled from 33.9 to 71.0 min (110%, *p* < 0.001) when no bout criterion was applied.

### 3.7 Plain and weighted variables

Both plain and weighted variables can be retrieved. Plain variables consist of an average of the collected data. Weighted variables are weighted 5:2 of data collected during the week and the weekend. We found no statistically significant difference in results on group level between using plain variables (mean 34.0 [SD 26.6] min/day) or weighted MVPA‐variables (mean 33.9 [SD 26.5] min/day, *p* = 0.24). Although, for some individuals there were differences up to 8.6 min of MVPA/day, varying in both directions. Neither did we find any statistically significant differences between plain and weighted variables for inactivity/day (mean 698 [SD 179] min and mean 698 [SD 179] min, *p* = 0.34) nor for LPA/day (mean 34.6 [SD 25.9] min and mean 34.7 [SD 25.9] min, *p* = 0.79).

## 4 DISCUSSION

Accelerometers collect physical activity data in an objective way; however, decisions during data processing can influence the output, as was shown in our data. For example, minutes unbouted MVPA was twice as high as when an 80% 1‐min bout criterion was applied. Historically, unbouted MVPA‐output has been used, but nowadays a bout criterion as in for example Whitehall II study^11^ is becoming more common. Further, MVPA/day were doubled when it was retrieved from GGIR part2 instead of part5, which indicates that the MVPA‐algorithms differs considerably. To our knowledge, part5 is the most commonly used output, for this type of study. We also found that the epoch‐length can affect the output. For example, minutes of MVPA/day and inactivity/day became lower when we changed the epoch‐length from 5 to 1 s, while LPA/day became higher.

It may be appealing to compare the output from GGIR with the historically common output from ActiLife to examine differences between the methods. This must however be made with caution as the default settings vary between the methods, such as epoch‐length and bout criteria. Nonetheless, Montoye et al.^14^ developed cut‐points for the nondominant wrist to apply when using ActiLife for data processing, and included a comparison between the methods. They found that using the output from GGIR lead to a lower level of misclassification, compared to when using the output from ActiLife.

There are situations when the default settings may be replaced with alternatives. For example, other cut‐points may be considered for persons with disabilities that affect their movement pattern. We only included adults without disabilities. Our suggestions and recommendations are thus primarily for this type of population. Further, the epoch‐length should be the same as when the applied cut‐points were developed, that is, if the cut‐points are changed, the epoch‐length should be changed accordingly.^1^

### 4.1 Placement of the accelerometer

We used the nondominant wrist, since it is the most common placement and therefore facilitates comparisons between studies.^2^ According to some authors, movements from the dominant wrist are on average 10% higher compared to movements from the nondominant wrist,^13^ while others mean that there is no difference.^16^ Using the ActiGraph GT3X+, Buchan et al.^17^ found an 8.5% higher average acceleration of the dominant wrist, compared to the nondominant wrist. This is in accordance with results from the consumer‐based activity monitor Fitbit.^18^ There is however no consensus on what wrist provides the best estimate of habitual total activity over several days.^3^ Even though the cut‐points for the dominant wrist are not supposed to be applied for accelerometer‐data collected using the nondominant wrist, our results illustrate the differences between the cut‐points, and stress the importance of instructing the participants on what wrist to wear the accelerometer.

### 4.2 Sampling frequency

As sampling frequency must be decided a priori, we decided to use 80 Hz in accordance with the NHANES‐study.^2^ This is a compromise between high sampling frequency and storage capacity. Frequencies between 30 and 100 Hz are available when using ActiGraph wGT3X‐BT,^7^ in contrast to previous versions that automatically collected data at 10 or 30 Hz.^19^

Previous studies have shown that sampling frequency affects the output when analyzed with ActiLife, especially for high intensity activities.^1^, ^19^ However, data collected at a variety of sampling frequencies can be processed using GGIR, since data is normalized by averaging into epochs as the first step of data processing.^4^, ^8^ While few studies have explored the impact of sampling frequency on the output in GGIR, Clevenger et al.^20^ conducted a comparison between data sampled at 30 Hz with data sampled at 100 Hz. They observed differences in the output both from ActiLife and GGIR. Even though further examination of the effect of sampling frequency is needed, this strengthens the argument that output collected and processed at different sampling frequency should be compared with caution.

Sampling frequency has implications far beyond data storage and downstream calculations. Fundamentally, the sampling frequency determines the range of movement frequencies that can be captured. Thus, it important to understand and consider the normal range of human movement frequencies. With this in mind, and since we do not know what processing methods the future holds, the best available recommendation is to use the highest possible sampling frequency, that is, 100 Hz if using the ActiGraph wGT3X‐BT.^1^ A lower frequency may be considered if the measurement period is very long (e.g., several weeks), due to storage limitations.^7^

### 4.3 MVPA‐cut‐point

We used the default MVPA‐cut‐point for the nondominant wrist (100 m*g*).^8^ This cut‐point or the cut‐point developed by Hildebrand et al.^12^ (100.6 m*g*), are usually recommended.^1^ However, these two cut‐points may be confused; as the former is often applied, but the latter is commonly referred to. Nevertheless, our study indicated that this may not be a major problem, since the difference in MVPA/day was marginal. However, the latter cut‐point is developed using 1 s epoch‐length compared to the default 5 s epoch‐length. When we changed the epoch‐length accordingly, we found statistically significantly lower MVPA/day by almost 20% (6.6 min), which can be considered clinically significant. Further, da Silva et al.^21^ also used the default cut‐point when processing accelerometer‐measurements from almost 9000 participants in three Brazilian cohort studies. The argument used was that it was within the “range of acceleration values corresponding to walking” and “a round number so as not to give the impression of over‐precision.”

Even though cut‐points are applied post data collection, it may be worth to consider MVPA‐cut‐point already beforehand, since a general recommendation is to keep all other parameters in accordance with when the cut‐point was developed.^1^ For example, if a MVPA‐cut‐point was developed using an accelerometer worn on the nondominant wrist and a sampling frequency of 100 Hz, these parameters are recommended to be constant when applying the cut‐point again.^1^

### 4.4 Definition of a valid day, valid week and wear‐time protocol

The number of valid days needed for an individual's physical activity assessment depends on requirements on data accuracy, which in turn, depends on compliance as well as day‐to‐day variation within the individuals. If all participants wear the device continuously, there is no need to have a longer wear‐time protocol than what is defined as a valid week, though some extra time is often added. The measurement period normally varies from 1 day up to several weeks, and a common strategy is to ask the participants to wear the accelerometer for seven consecutive days, to include both weekdays and weekend days.^1^, ^15^ Further, compliance increases if the participants are instructed to wear the accelerometer continuously, instead of only during waking hours.^1^

We found no difference in MVPA/day when we changed the criteria for neither a valid day nor a valid week. In general, more hours and days of valid wear‐time may provide a more correct assessment of an individual's physical activity level, however, a higher wear‐time criterion may lower the number of participants that fulfills this criterion. In other words, the definition of a valid day and week can in practice be a trade‐off between accuracy and sample size.^1^

### 4.5 Strengths and limitations

The field of accelerometry is not yet standardized, and our results clearly indicates that decisions made during this process can have an influence on the output.

Although technological advances continue to develop the field, practical tips for data collection and processing will likely remain similar. It is a strength that we have used an ActiGraph‐accelerometer. This is the most commonly used device to objectively measure physical activity, and >50% of studies from different parts of the world report using an accelerometer from ActiGraph.^22^ Further, it is also a strength that we used GGIR for data processing, as it harmonizes data collected using different accelerometers and therefore facilitates comparisons between studies. GGIR is a simple and cost‐effective method, which makes it possible for other researchers to reproduce similar analyses, and has so far been used in more than 170 peer‐reviewed scientific publications.^23^

Some decisions must be made before the data collection, for example sampling frequency and placement of the accelerometer. Since we used collected data for our analyses, we could not test the influence of those choices, which is a limitation. Further, adults with diabetes have a higher risk of foot ulcers compared to the general population. This can impede walking, making the choice of population less optimal. However, since one of our exclusion criteria were: not being able to walk, we believe that our results can be generalized to the population of adults without diabetes. Another limitation is that our sample size was small and we were not able to run stratified analyses.

In conclusion, decisions during data processing can have a major influence on the output. A general recommendation to keep in mind, is that if default settings are changed, the influence on the output of interest (e.g., MVPA/day) may be examined. Further, we strongly encourage to report decisions made during data processing, to facilitate comparisons between studies.

## 5 PERSPECTIVE

Accelerometers have made collection of objective physical activity data possible, also in large studies. To date, the field of accelerometry and the many decisions that must be made during data processing and output interpretation are yet not standardized. The most exhaustive guidelines for data processing are found in the review by Migueles et al.^1^ However, their main focus was on data processing of hip‐worn accelerometers using a brand‐specific program, and not on utilization of raw data collected using wrist‐worn accelerometers. Therefore, we have compiled information on these specific decisions from several different sources, such as manuals for data processing,^7^, ^8^, ^10^ methodological studies^4^, ^9^, ^12^, ^16^, ^20^ as well as larger epidemiological studies^11^, ^21^ in combination with original tests on collected accelerometer‐data. We recommend that researchers combine these sources of information to stay updated within the field of accelerometry. Our results put these decisions into perspective, and indicate how these can influence the output, and in the extension, the conclusions drawn.

## AUTHOR CONTRIBUTIONS

All authors have read and approved the final version of the manuscript. The corresponding author Helén Eke had full access to all of the data in this study and takes complete responsibility for the integrity of the data and the accuracy of the data analysis.

## CONFLICT OF INTEREST STATEMENT

The authors declare no conflict of interest.

## ETHICS STATEMENT

The study was approved by the Regional ethical review board in Stockholm (2016/2041–31/2; 2016/99–32; 2017/1405–32; 2018/286‐32). All participants provided written informed consent.

## TRANSPARENCY STATEMENT

The lead author Helén Eke affirms that this manuscript is an honest, accurate, and transparent account of the study being reported; that no important aspects of the study have been omitted; and that any discrepancies from the study as planned (and, if relevant, registered) have been explained.

## Data Availability

Deidentified data that support the findings of this study are available from the corresponding author upon reasonable request. The data is not publicly available due to ethical restrictions.

## References

[1] Migueles JH , Cadenas‐Sanchez C , Ekelund U , et al. Accelerometer data collection and processing criteria to assess physical activity and other outcomes: a systematic review and practical considerations. Sports Med. 2017;47(9):1821‐1845. 10.1007/s40279-017-0716-0 28303543 PMC6231536

[2] Troiano RP , McClain JJ , Brychta RJ , Chen KY . Evolution of accelerometer methods for physical activity research. Br J Sports Med. 2014;48(13):1019‐1023. 10.1136/bjsports-2014-093546 24782483 PMC4141534

[3] LaMunion SR , Fitzhugh EC , Crouter SE . Challenges and opportunities related to the objective assessment of physical activity within U.S. health surveys. Ann Epidemiol. 2020;43:1‐10. 10.1016/j.annepidem.2020.01.011 32147321

[4] Migueles JH , Rowlands AV , Huber F , Sabia S , van Hees VT . GGIR: a research community–driven open source R package for generating physical activity and sleep outcomes from multi‐day raw accelerometer data. J Measure Phys Behav. 2019;2(3):188‐196. 10.1123/jmpb.2018-0063

[5] Montoye AHK , Moore RW , Bowles HR , Korycinski R , Pfeiffer KA . Reporting accelerometer methods in physical activity intervention studies: a systematic review and recommendations for authors. Br J Sports Med. 2018;52(23):1507‐1516. 10.1136/bjsports-2015-095947 27539504

[6] Bonn SE , Alexandrou C , Hjörleifsdottir Steiner K , et al. App‐technology to increase physical activity among patients with diabetes type 2—the DiaCert‐study, a randomized controlled trial. BMC Public Health. 2018;18(1):119. 10.1186/s12889-018-5026-4 29316905 PMC5761151

[7] ActiGraph . User Guide. ActiGraph wGT3X‐BT + ActiLife. 2019. https://6407355.fs1.hubspotusercontent-na1.net/hubfs/6407355/User%20Manuals/ActiGraph_wGT3X-BT_UserGuide_E.200.6003_Revision7.pdf

[8] Van Hees VT . Accelerometer data processing with GGIR. 2020. https://cran.r-project.org/web/packages/GGIR/vignettes/GGIR.html

[9] van Hees VT , Fang Z , Langford J , et al. Autocalibration of accelerometer data for free‐living physical activity assessment using local gravity and temperature: an evaluation on four continents. J Appl Physiol. 2014;117(7):738‐744. 10.1152/japplphysiol.00421.2014 25103964 PMC4187052

[10] Van Hees VT . Raw Accelerometer Data Analysis. 2020. https://cran.r-project.org/web/packages/GGIR/GGIR.pdf

[11] Menai M , van Hees VT , Elbaz A , Kivimaki M , Singh‐Manoux A , Sabia S . Accelerometer assessed moderate‐to‐vigorous physical activity and successful ageing: results from the whitehall II study. Sci Rep. 2017;8:45772. 10.1038/srep45772 28367987 PMC5377945

[12] Hildebrand M , van Hees VT , Hansen BH , Ekelund U . Age group comparability of raw accelerometer output from wrist‐ and hip‐worn monitors. Med Sci Spo Exer. 2014;46(9):1816‐1824. 10.1249/mss.0000000000000289 24887173

[13] Migueles JH , Cadenas‐Sanchez C , Rowlands AV , et al. Comparability of accelerometer signal aggregation metrics across placements and dominant wrist cut points for the assessment of physical activity in adults. Sci Rep. 2019;9(1):18235. 10.1038/s41598-019-54267-y 31796778 PMC6890686

[14] Montoye AHK , Clevenger KA , Pfeiffer KA , et al. Development of cut‐points for determining activity intensity from a wrist‐worn ActiGraph accelerometer in free‐living adults. J Sports Sci. 2020;38(22):2569‐2578. 10.1080/02640414.2020.1794244 32677510

[15] Aadland E , Ylvisåker E . Reliability of objectively measured sedentary time and physical activity in adults. PLoS One. 2015;10(7):e0133296. 10.1371/journal.pone.0133296 26192184 PMC4508000

[16] Buchan DS , McSeveney F , McLellan G . A comparison of physical activity from actigraph GT3X+ accelerometers worn on the dominant and non‐dominant wrist. Clin Physiol Funct Imaging. 2019;39(1):51‐56. 10.1111/cpf.12538 30058765

[17] Buchan DS , Boddy LM , McLellan G . Comparison of Free‐Living and laboratory activity outcomes from ActiGraph accelerometers worn on the dominant and Non‐Dominant wrists. Measur Phys Educat Exer Sci. 2020;24(4):247‐257. 10.1080/1091367X.2020.1801441

[18] Clevenger KA , Molesky MJ , Vusich J , Montoye AHK . Free‐Living comparison of physical activity and sleep data from fitbit activity trackers worn on the dominant and nondominant wrists. Measure Phys Educat Exerc Sci. 2019;23(2):194‐204. 10.1080/1091367X.2019.1577737

[19] Brønd JC , Arvidsson D . Sampling frequency affects the processing of actigraph raw acceleration data to activity counts. J Appl Physiol. 2016;120(3):362‐369. 10.1152/japplphysiol.00628.2015 26635347

[20] Clevenger KA , Brønd JC , Arvidsson D , et al. Impact of ActiGraph sampling rate and intermonitor comparability on measures of physical activity in adults. J Measure Phys Behav. 2021;4(4):287‐297. 10.1123/jmpb.2021-0016

[21] da Silva IC , van Hees VT , Ramires VV , et al. Physical activity levels in three Brazilian birth cohorts as assessed with raw triaxial wrist accelerometry. Int J Epidemiol. 2014;43(6):1959‐1968. 10.1093/ije/dyu203 25361583 PMC4276065

[22] Wijndaele K , Westgate K , Stephens SK , et al. Utilization and harmonization of adult accelerometry data: review and expert consensus. Med Sci Spor Exer. 2015;47(10):2129‐2139. 10.1249/MSS.0000000000000661 PMC4731236 25785929

[23] Van Hees VT . Peer‐reviewed academic publications using GGIR. 2022. https://github.com/wadpac/GGIR/wiki/Publication-list

